# Effective inhibition of melanoma tumorigenesis and growth via a new complex vaccine based on NY-ESO-1-alum-polysaccharide-HH2

**DOI:** 10.1186/1476-4598-13-179

**Published:** 2014-07-28

**Authors:** Meng Li, Huashan Shi, Yandong Mu, Zichao Luo, Hailong Zhang, Yang Wan, Dongmei Zhang, Lian Lu, Ke Men, Yaomei Tian, Xiaozhe Wu, Xiaoyan Liu, Ying Pan, Yingzi Fan, Chaoheng Yu, Bailing Zhou, Rong Xiang, Xiancheng Chen, Li Yang

**Affiliations:** 1 State Key Laboratory of Biotherapy / Collaborative Innovation Center for Biotherapy, West China Hospital, West China Medical School, Sichuan University, Chengdu, PR China; 2 Department of Stomotology, Sichuan Academy of Medical Science & Sichuan Provincial People’s Hospital, Chengdu, PR China; 3 Institute of Biomedical Engineering, School of Ophthalmology & Optometry and Eye Hospital, Wenzhou Medical College, Wenzhou, PR China; 4 Department of Immunology, Nankai University School of Medicine, Tianjin, PR China

**Keywords:** Alum, Polysaccharide, HH2, Adjuvant complex, NY-ESO-1 protein-based vaccines, Melanoma, Cross-presentation

## Abstract

**Background:**

A safe and effective adjuvant plays an important role in the development of a vaccine. However, adjuvants licensed for administration in humans remain limited. Here, for the first time, we developed a novel combination adjuvant alum-polysaccharide-HH2 (APH) with potent immunomodulating activities, consisting of alum, polysaccharide of *Escherichia coli* and the synthetic cationic innate defense regulator peptide HH2.

**Methods:**

The adjuvant effects of APH were examined using NY-ESO-1 protein-based vaccines in prophylactic and therapeutic models. We further determined the immunogenicity and anti-tumor effect of NY-ESO-1-APH (NAPH) vaccine using adoptive cellular/serum therapy in C57/B6 and nude mice. Cell-mediated and antibody-mediated immune responses were evaluated.

**Results:**

The APH complex significantly promoted antigen uptake, maturation and cross-presentation of dendritic cells and enhanced the secretion of TNF-α, MCP-1 and IFN-γ by human peripheral blood mononuclear cells compared with individual components. Vaccination of NAPH resulted in significant tumor regression or delayed tumor progression in prophylactic and therapeutic models. In addition, passive serum/cellular therapy potently inhibited tumor growth of NY-ESO-1-B16. Mice treated with NAPH vaccine produced higher antibody titers and greater antibody-dependent/independent cellular cytotoxicity. Therefore, NAPH vaccination effectively stimulated innate immunity, and boosted both arms of the adaptive humoral and cellular immune responses to suppress tumorigenesis and growth of melanoma.

**Conclusions:**

Our study revealed the potential application of APH complex as a novel immunomodulatory agent for vaccines against tumor refractory and growth.

## Background

NY-ESO-1 is a member of cancer/testis antigen expressed in multiple types of tumors, but not in normal adult tissues except for germ cells in testes and ovaries [1-3]. To date, a large number of major histocompatibility complex (MHC) class I- and class II-restricted NY-ESO-1 epitopes have been identified [4-7]. Studies have demonstrated that coordinated humoral and cellular immune responses against NY-ESO-1 occurred frequently in patients bearing NY-ESO-1-positive tumors [6,8-10]. The relatively high immunogenicity and tissue distribution make NY-ESO-1 a promising candidate antigen for the development of tumor vaccines.

Vaccination with protein or peptide alone is insufficient to elicit an effective immune response. Adjuvant plays an important role in modifying the Th1/Th2 balance and increasing the magnitude and durability of adaptive immunity of subunit vaccines [11-13]. However, adjuvants licensed for human administration remain limited. Alum, the adjuvant for vaccine first approved by the United States Food and Drug Administration, is still the most commonly used adjuvant [14,15]. Many studies have demonstrated its depot effect on antigens, resulting in its retention at the immunization site to prolonged antigen exposure [16,17]. The administration of alum with protein or peptide elicits a stronger antibody-mediated immune response with a Th2 bias [18,19]. Generation of a cellular immune response stimulated by vaccines with alum alone as an adjuvant is less well documented. As a poor CD8^+^ T cell adjuvant, alum alone is not suitable for therapeutic vaccines. Effective cytotoxic T-cell response may be stimulated in the presence of other additional adjuvants. Thus, the search for new, safe and potent complex adjuvant with the ability to enhance both the humoral and cellular immune responses remains a priority for the development of future vaccines.

Numerous polysaccharides (PSs) have been reported to act as potent immunomodulators with the function of stimulating innate and adoptive immune responses [20-22]. Many studies have demonstrated that polysaccharides in combination with antitumor monoclonal antibodies (mAbs) result in significant inhibition of tumor growth and enhancement of long-term survival [20-22]. β-glucans are polysaccharides extracted from yeast, barley, mushroom, seaweed, bacteria, and oat [20,21,23,24]. Soluble β-glucans can prime neutrophils, macrophages, and NK cells for their cytotoxicity against iC3b-opsonized tumor cells resulting from the complement activation by antitumor mAbs [25,26]. Dendritic cells (DCs) activated by bacterial β-glucan Curdlan can convert Treg cells into IL-17-producing T cells and simultaneously stimulate Th1, Th17 and cytotoxic T lymphocyte priming and differentiation [27]. The immunomodulatory function of the bacterial polysaccharide was further illustrated by a study demonstrating that β-glucan enhances antitumor immune responses by regulating the differentiation and function of Treg cells and monocytic myeloid-derived suppressor cells [28]. In particular, with a short production cycle, low production cost and no limitation of season and geographical restriction, bacterial extracellular polysaccharides may be a good source that can be exploited for immunotherapy. However, so far, little is known about bacterial polysaccharides as an adjuvant. Recently, for the first time, we have studied the immunomodulatory effect of alum-PS complex (AP). The AP complex can stimulate effective antigen-specific humoral immune response, but has limited protective cellular immune responses. Thus, improvements are required before the antitumor vaccine becomes more effective against cancer.

HH2 (VQLRIRVAVIRA-NH_2_) is a short synthetic innate defense-regulator peptide (IDRs). The immunomodulatory functions of IDRs include indirect or direct recruitment of DC, neutrophils and macrophages, modulation of DC and macrophage differentiation, stimulation of production of chemokines (monocyte chemoattractant protein-1, MCP-1) and cytokines (IL-8, IFN-γ), limitation of potentially harmful inflammation mediated by bacterial products, and adjuvant activity stimulating innate immune response and promoting a vigorous adaptive response [29-31]. The development of natural host defense peptides has been problematic due to their hemolytic activity, cytotoxicity toward mammalian cells and stimulation of degranulation of mast cells [32,33]. With the advantage of more optimized immunomodulatory activities and low cytotoxicity over natural IDRs, the synthetic mimics (e.g. HH2) of innate defense regulators may be attractive candidates for immunotherapy.

In the present study, we developed an alum-polysaccharide-HH2 (APH) complex as a novel adjuvant, and examined the antitumor effect of the NY-ESO-1 protein vaccine with the APH adjuvant in a melanoma model. The APH complex adjuvant dramatically enhanced the quantitative strength of both NY-ESO-1 specific humoral and cellular immune responses against melanoma formation and progression. Therefore, our findings indicate that APH is a new promising immunomodulatory agent for vaccines against tumor refractory and growth.

## Results

### The PS-HH2 complexes induce chemokines in peripheral blood mononuclear cells

Preliminary studies demonstrated that HH2 and the TLR ligands are potent inducers of MCP-1, a chemokine that is chemotactic for monocytes/macrophages, NK cells, and neutrophils [30]. Some cytokines are important in innate immunity, such as TNF-α and IFN-γ. Thus, MCP-1, TNF-α, and IFN-γ were selected to screen for the development of immunostimulatory agents. This synergistic effect was calculated as the total release of chemokines induced by a PS-HH2 complex divided by the additive release of chemokines induced by the individual complex components. A cut-off level of ≥2 was selected to indicate synergy. As shown in Additional file 1: Figure S1A, the 1:2 ratio of PS: HH2 resulted in a maximal IFN-γ induction (1352 ± 95 pg/ml) among these groups. The potent induction of TNF-α expression *in vivo* showed an increasing trend with an increasing concentration of HH2. Among the PS-HH2 complexes, 1:4 and 1:2 (wt/wt) ratios of PS: HH2 resulted in a most potent induction of TNF-α expression (699 ± 48 pg/ml and 651 ± 104pg/ml, respectively) in vitro and independently showed a synergistic effect of 2.1 ± 0.1 and 2 ± 0.2, respectively (Additional file 1: Figure S1B). Similarly, 1:4 and 1:2 (wt/wt) ratios of PS: HH2 resulted in a most potent induction of MCP-1 expression (16064 ± 2051 pg/ml and 13725 ± 1480 pg/ml, respectively). There was no significant difference between the two groups (Additional file 1: Figure S1C). Since safety is as important as potency for an adjuvant, we further investigated the potential cytotoxic effect of the PS-HH2 complexes by monitoring the release of hemoglobin from red blood cells and lactate dehydrogenase (LDH) from peripheral blood mononuclear cells (PBMCs). Our data showed that the tested PS-HH2 complexes resulted in minimal or no release of hemoglobin or LDH from red blood cells (RBCs) (Additional file 1: Figure S1D) and PBMCs (Additional file 1: Figure S1E), respectively. Thus, 1:2 (wt/wt) PS: HH2 formulation was chosen to further examine its adjuvant effect for NY-ESO-1 below.

### NAPH vaccine inhibits tumor growth in a melanoma model

**Figure 1:**
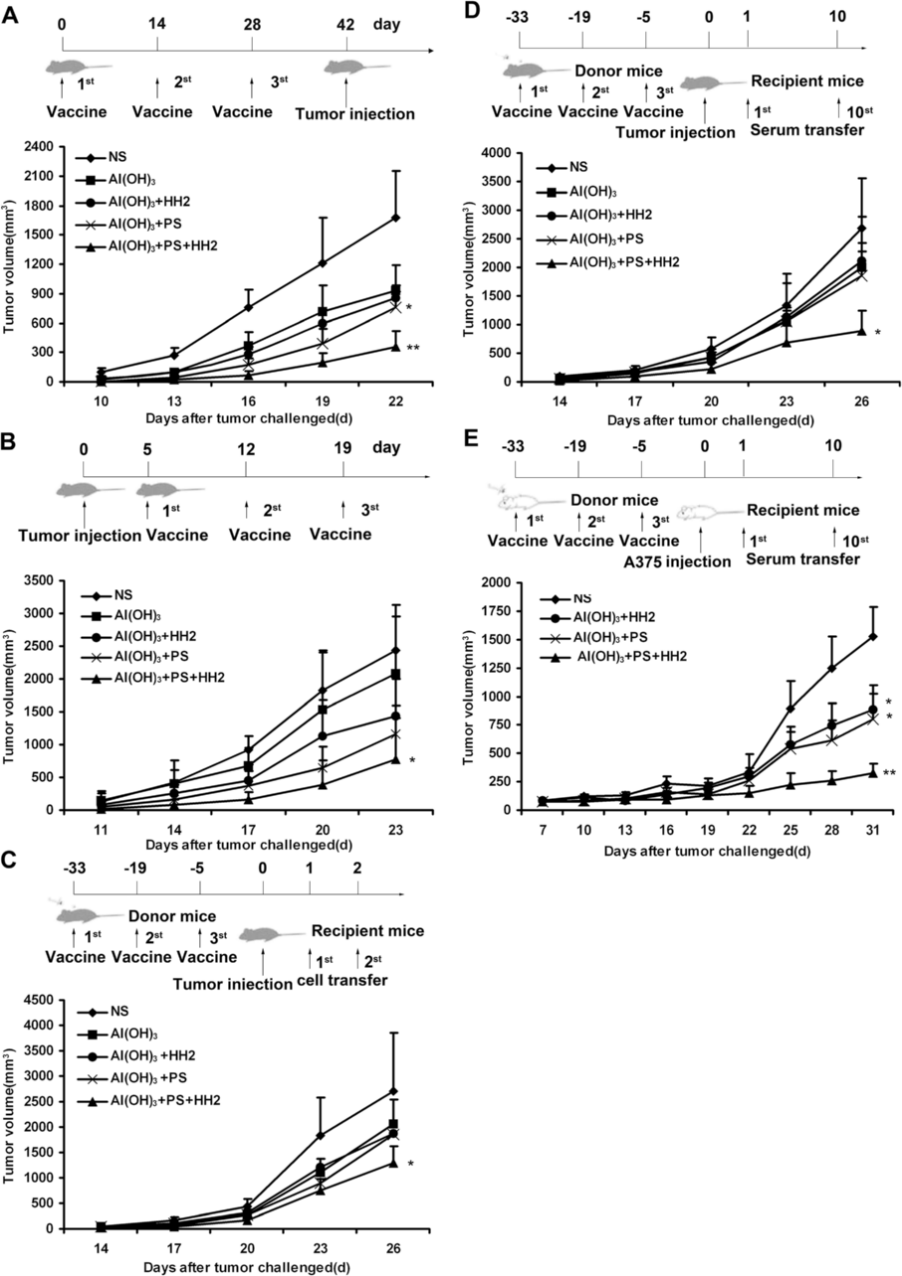
**NAPH vaccine inhibits the melanoma tumorigenesis and growth. A**, C57/B6 mice (n = 12) were treated with different vaccines (on Days 0, 14 and 28) and challenged with 2 × 10^5^ NY-ESO-1-B16 cells (on Day 42) in a prophylactic model. **B**, NY-ESO-1-B16-bearing mice (n = 12) were treated with vaccines (on Days 5, 12 and 19) in a therapeutic model. **C, D**, Adoptive transfer of cells or serum from NAPH-treated mice induced tumor regression in recipient mice. Donor splenocytes (2 × 10^7^) were injected i.v. into recipient mice for 2 consecutive days (on Days 1 and 2). In serum adoptive therapy, tumor-bearing mice were injected i.v. with 0.1 ml of the sera from Days 1 to 10. **E**, Adoptive transfer of serum from immunized-Balb/c mice inhibited A375 melanoma growth in a nude mouse model. * *P* < 0.05; ** *P* < 0.005.

In the prophylactic model (Figure 1A and Additional file 2: Tables S1 and S2), the NY-ESO-1-B16 melanoma grew rapidly in control mice, with mean tumor volume of approximately 1675 mm^3^ by Day 22. In contrast, the tumor growth was significantly inhibited in the NY-ESO-1-alum-PS (NAP) group (*P* < 0.05 versus control). Of more interest, the tumor growth was significantly delayed in the NAPH group, in which nearly half of these mice were still tumor-free by Day 22 (*P* < 0.005 versus control). These results indicated that vaccination of mice with the NAPH vaccine induced an effective NY-ESO-1-specific antitumor immune response, which was sufficient to suppress the tumorigenesis and growth of NY-ESO-1-B16 melanoma.

In the therapeutic model (Figure 1B and Additional file 2: Tables S1 and S2), mice in the control group developed large tumors (mean volume, 2431 mm^3^) by Day 23 after NY-ESO-1-B16 melanoma cells were inoculated into mice. The mean volumes of melanoma in the NY-ESO-1-alum (NA), NY-ESO-1-alum-HH2 (NAH), NAP, and NAPH group were 2079, 1434, 1155, and 773 mm^3^, respectively. The tumor growth in the NS or NA-immunized groups was progressive, but in NAPH-treated group was significantly retarded (*P* < 0.05 versus control).

The anti-tumor effect of the NAPH vaccine was further studied in an adoptive cellular/serum therapy model. As shown in Figure 1C and Additional file 2: Tables S1 and S2, the mean volumes of melanoma in mice transferred with splenocytes from NS, NA, NAH, NAP and NAPH groups were 2699, 2052, 1879, 1858, and 1282 mm^3^, respectively. Tumor growth of NAPH-treated mice was significantly inhibited after passive cellular therapy (*P* < 0.05 versus control).

To determine whether the serum was sufficient to mediate the antitumor response, serum from the vaccinated mice was passively transferred to recipient C57/B6 mice for 10 consecutive days one day after the tumor challenge (Figure 1D). C57/B6 mice receiving serum from naïve mice all succumbed to tumors. In contrast, serial passive transfer of serum from the NAPH group significantly inhibited NY-ESO-1-B16 tumor growth (*P* < 0.05 versus control).

To further study the effect of passive serum therapy on A375 human melanoma model, A375-bearing mice were subjected to passive serum therapy, as described above, with serum from treated or naïve Balb/c mice (Figure 1E). Immune serum from the NAPH group caused retardation or regression of A375 tumor xenografts. Upon sacrifice, the mean tumor volumes from mice treated with NAPH or non-immune sera were 328 mm^3^ and 1521 mm^3^, respectively, with a significant difference (*P* < 0.005) (Additional file 2: Tables S1 and S2).

No marked toxicity, including weight loss, ruffed fur, diarrhea, and anorexia, was observed during the entire vaccination in the preventive model. To evaluate the potential toxic effects on vital organs in treated mice, hematoxylin-eosin staining (HE staining) was performed. There were no obvious pathological changes in the heart, liver, spleen, lung and kidney of the treated mice (Additional file 3: Figure S2).

### NAPH vaccine effectively stimulates an innate immune response

**Figure 2:**
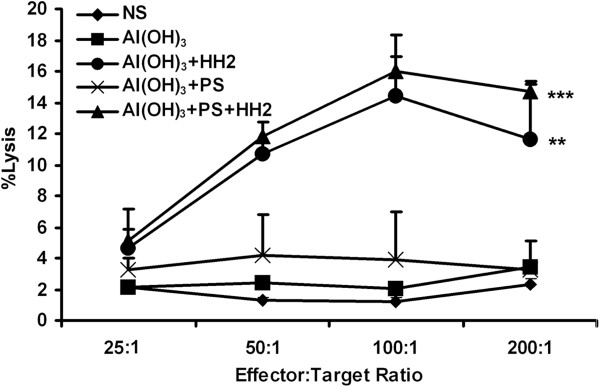
**NAPH vaccine stimulates the innate immune response.** 48 hours after NAPH injection, the mice were sacrificed. A standard 4 h NK assay against YAC-1 targets revealed that the NK cytotoxicity in the NAH group was higher than that in the NS, NA and NAP groups; in particular, NAPH-treated mice had the greatest cytotoxic activity against YAC-1 cells among all groups (* *P* < 0.05; ** *P* < 0.005).

The immunostimulatory effect of the NAPH vaccine on innate immunity was examined by measuring the NK-cell activity assay 48 h after the first immunization. As shown in Figure 2, NK-cells from control mice were unable to lyse YAC-1 cells when total splenocytes were used as effectors; there was no significant difference between the NS group and NA or NAP group in NK-cell-mediated cytolysis of YAC-1 cells, while the cytotoxicity in the NAH group was higher than that in the above-mentioned groups (*P* < 0.005). Splenocytes isolated from NAPH-treated mice had a greater cytotoxic activity against YAC-1 cells than that from the NAP group (*P* < 0.001). Therefore, the NAPH vaccine was able to effectively stimulate innate immunity to eradicate MHC-I-deficient tumors *in vivo*.

### NAPH vaccine enhances antigen-specific antibody immune response

**Figure 3:**
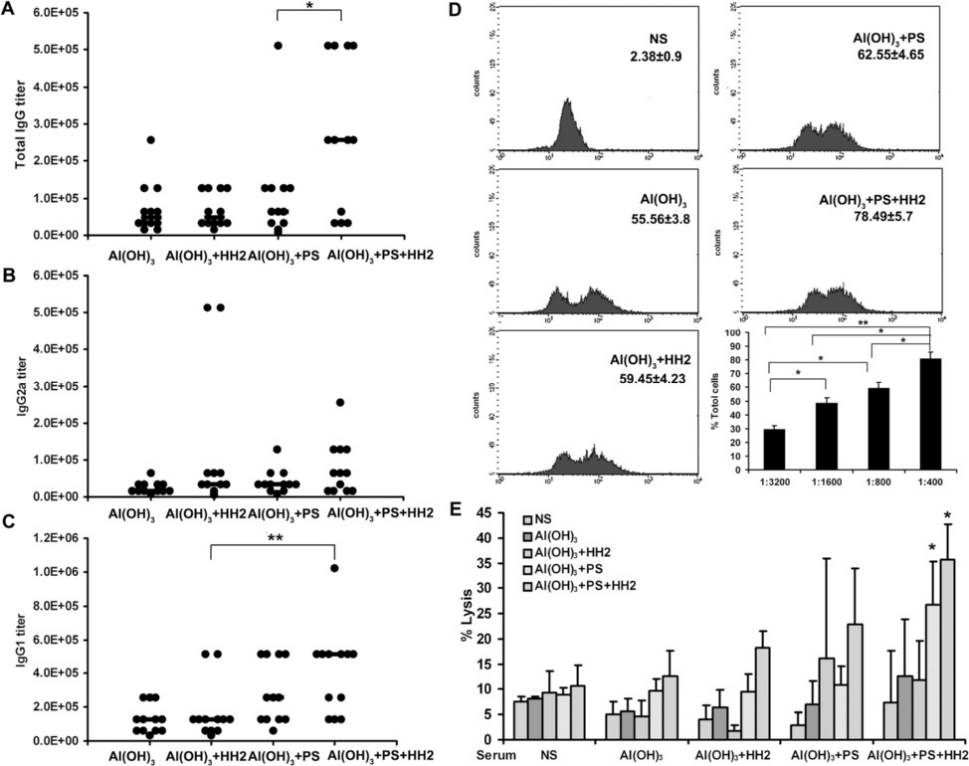
**NAPH increases NY-ESO-1-specific antibody immune response. A**, The titers of anti-NY-ESO-1 total IgG were measured using ELISA. Dot, titer of each mouse. **B**, **C**, The titers of anti-NY-ESO-1 IgG2a/IgG1 titers in the treated groups were measured. **D**, FACS analysis using NY-ESO-1-B16 cells to detect NY-ESO-1-specific IgG antibodies (n = 3 mice, pooled composite results are shown). **E**, Antibody-dependent cellular cytotoxicity was determined using the ^51^Cr-release assay. NY-ESO-1-B16 cells were labeled with ^51^Cr and coated with sera from control or treated mice. Splenocytes from untreated or treated mice were used as effectors. The assay was run in three replicates.

Sera from naïve or treated mice were analyzed using ELISA for the presence of total NY-ESO-1-specific IgG and different IgG subtypes one week after the third immunization. NAPH-immunized mice developed significantly higher titers of NY-ESO-1-specific IgG than other groups (*P* < 0.05) (Figure 3A). Although there was an increasing trend for IgG2a titer in NAPH group compared with other groups, these differences did not achieve statistical significance (*P* > 0.05) (Figure 3B). Sera obtained from the NAPH group showed substantial higher titer of IgG1 than that from other groups (*P* < 0.005) (Figure 3C). Antibody binding to natural NY-ESO-1 expressed by NY-ESO-1-B16 melanoma cells in the NAPH group was the highest among the groups (*P* < 0.05). The serum binding positive index of NY-ESO-1-B16 increased with a decreased serum dilution ratio (Figure 3D).

To investigate whether the elicited antibody mediates antibody-dependent cell-mediated cytotoxicity (ADCC) activity (Figure 3E), NY-ESO-1-B16 melanoma cells were utilized as target cells after coating with sera from different groups. Sera from control showed no lysis of NY-ESO-1-B16 melanoma cells. Sera from NAPH-treated mice induced a significant ADCC particularly when the effectors were derived from the NAPH group (*P* < 0.05).

### Induction of potent cytotoxic T lymphocytes (CTL) responses plays a critical role in anticancer activity of NAPH vaccine

**Figure 4:**
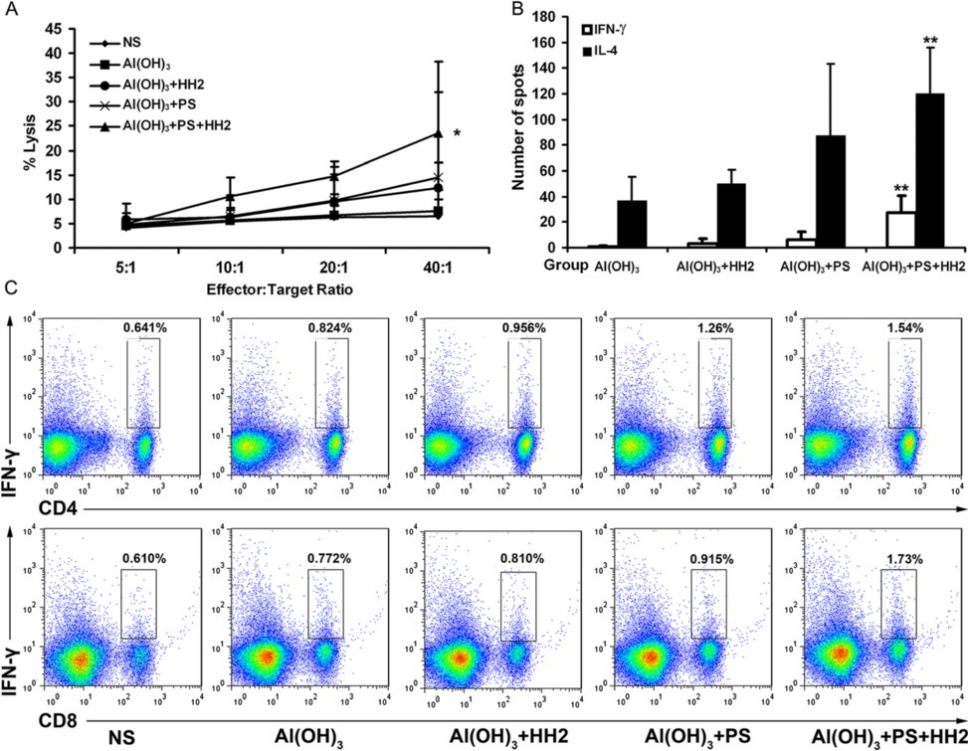
**NAPH vaccination elicits robust NY-ESO-1-specific cellular response *in vitro*. A**, The cytotoxicity of splenocytes against NY-ESO-1-B16 cells was examined in a 4-h ^51^Cr-release assay. Points, mean of triplicate samples; bars, SD. **B**, IL-4/IFN-γ ELISPOT responses were performed at one week post-immunization (n = 3, three replicates). **C**, Intracellular staining of IFN-γ in CD4^+^ and CD8^+^ T cells. Similar results were obtained from three independent experiments (*P < 0.05; ** *P* < 0.005).

To assess the cell-mediated immune response stimulated by the NAPH vaccine, CTL activity was performed using the ^51^Cr release assay. Splenocytes were separated and stimulated with 10 μg/ml NY-ESO-1 for 16 h and used as effectors. CTL assays (Figure 4A) were validated by the ratio of spontaneous release to maximum release. These effectors in NAPH showed 2.2-fold more tumor-killing activity than that in the NA group, and 1.2-fold more than that in the NAP or NAH groups (*P* < 0.05 versus control). In the ELISPOT assay, the number of spot-forming cells (SFCs) after *in vitro* stimulation with NY-ESO-1 is shown in Figure 4B. There was a more pronounced increase in SFCs secreting IFN-γ/IL-4 in the NAP-immunized group than the NA or NAH group (*P* < 0.05). The number of SFCs secreting IFN-γ instead of IL-4 in the NAPH group was 3.1-fold greater than that of the NAP group (*P* < 0.05). To further confirm this observation, splenocytes obtained from naïve or treated mice were stimulated with NY-ESO-1 protein, and the frequency of IFN-γ-secreting CD4^+^/CD8^+^ T cells was measured via flow cytometry (Figure 4C). The mean fluorescence intensity of IFN-γ expression by CD4^+^ and CD8^+^ T cells was the highest in the NAPH group (*P* < 0.05 versus NS and NA groups).

### The NAPH vaccine increases infiltrating lymphocytes in tumors

**Figure 5:**
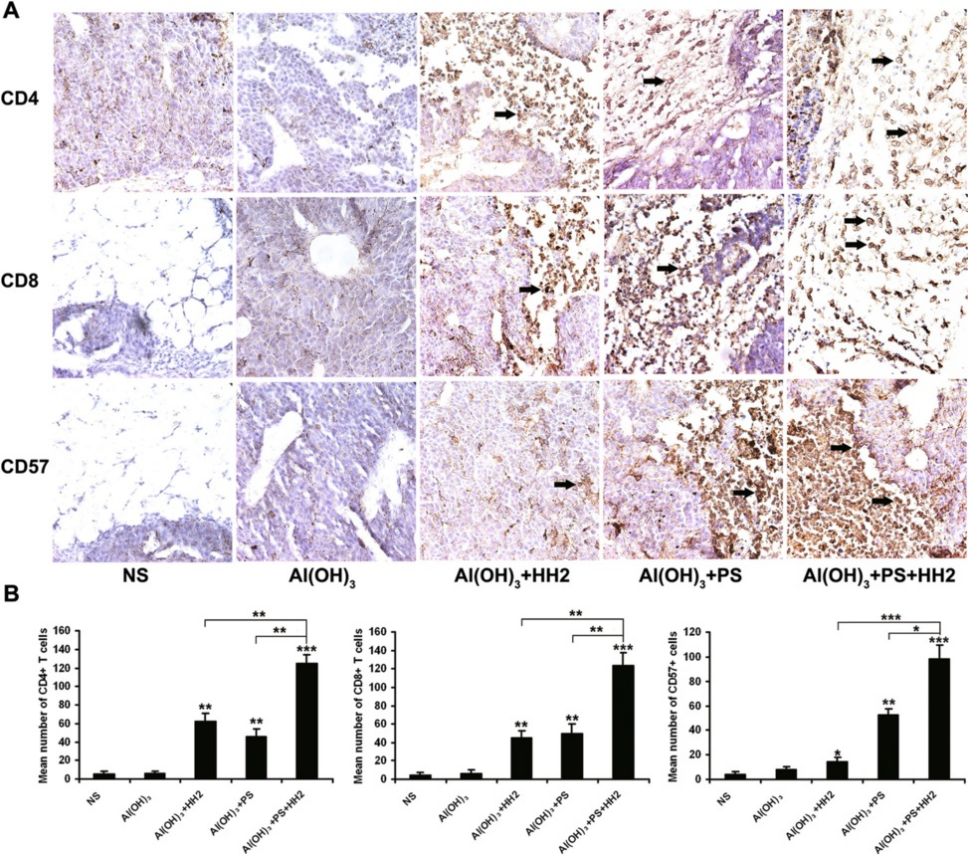
**Immunohistochemistry analyses were performed to study infiltrating lymphocytes (TILs) in tumors. A**. Tumor tissue sections were separately incubated with anti-CD4, anti-CD8 and anti-CD57 mAbs overnight at 4°C. Sections were then incubated with biotinylated anti-mouse antibody and streptavidin-biotinylated peroxidase complex. **B**. Intensity of lympocyte infiltration in the tumors was determined by the mean positive cell counts in the dermis around the B16-NY-ESO-1 tumor per field (10 randomly selected high power fields/slide). For each site, 3 pathologists performed a blind read of the glass slides.

Tumor infiltrating lymphocytes (TILs), a primary immune component infiltrating solid tumors, are considered to be the manifestation of the host antitumor reaction. Immunohistochemistry analyses were performed to study TILs in tumors. The intensity of infiltration by CD4, CD8 and CD57 lymphocytes was studied (Figure 5A). There was a significant increase in the intensity of infiltration of CD4^+^ and CD8^+^ T cells in tumors from the NAH and NAP groups compared with the NS and NA groups (*P* < 0.005). NK cell infiltration in tumors from the NAP group was higher than that from the NS and NA groups (*P* < 0.005). Importantly, a large number of CD4^+^, CD8^+^ T cells and NK cells infiltrated were observed in the tumors from the NAPH group, compared with the NS group (*P* < 0.001) (Figure 5B).

### The PS-HH2 complex promotes antigen uptake and cross-presentation of dendritic cells

**Figure 6:**
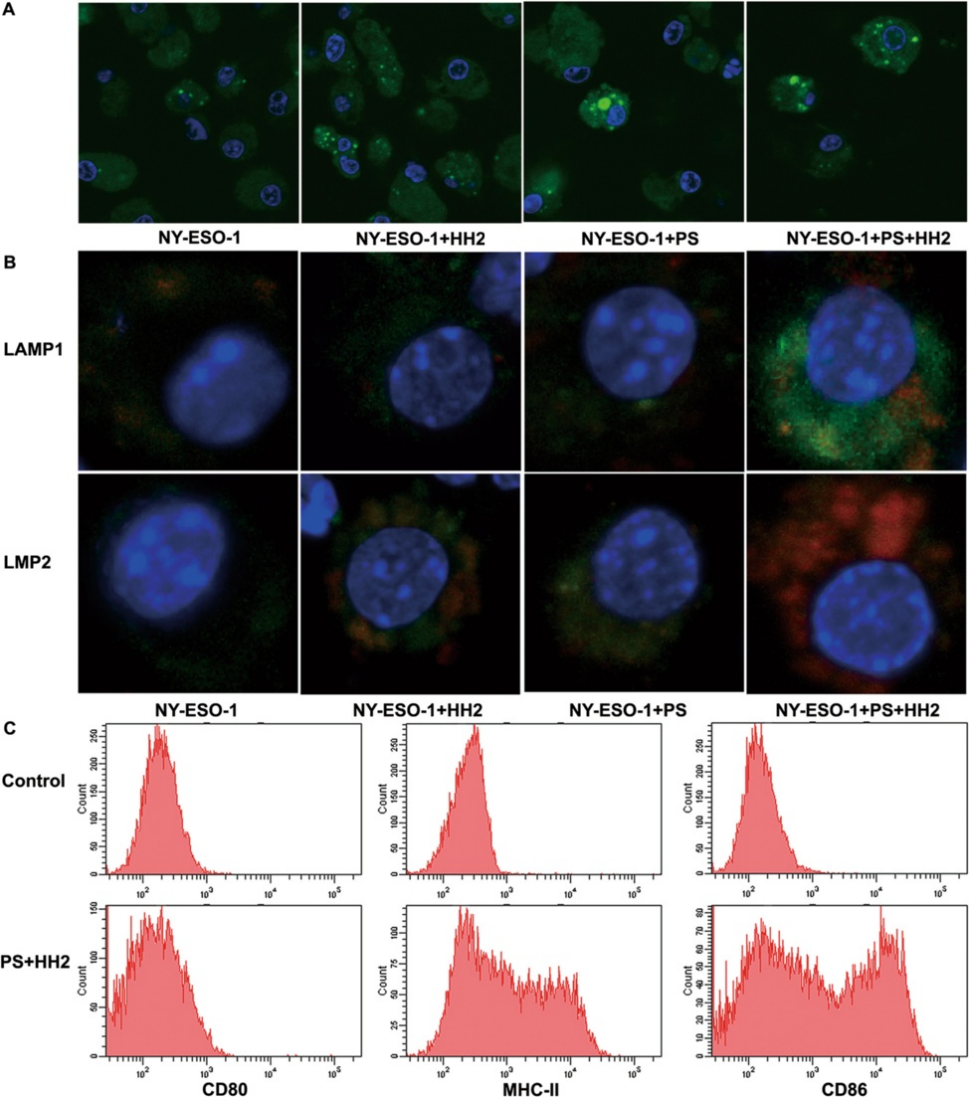
**PS-HH2 complex adjuvant promotes the antigen uptake and cross -presentation and maturation of DCs. A**, DC were incubated with NHS-conjugated native NY-ESO-1 or protein adjuvant complex for 3 h, fixed, stained with DAPI and analyzed via confocal laser microscopy. **B**, DCs were incubated with Alexa Fluor-labeled NY-ESO-1 or NY-ESO-1/complex adjuvant for 3 h, fixed, permeabilized, blocked and stained with mouse FITC-anti-LAMP1 or FITC-anti-LMP2 antibody, respectively. DNA was stained with DAPI and analyzed via confocal laser microscopy. **C**, Analysis of surface maturation markers of DCs stimulated with PS-HH2 for 24 h.

To investigate the effect of complex adjuvant on antigen uptake of DCs, we directly visualized the uptake capacity of NY-ESO-1. As shown in Figure 6A, the mean fluorescence intensity values of NY-ESO-1 alone, NH, NP and NAPH stimulated DCs were 9.5, 18.9, 64.0 and 118.0, respectively. There was a significantly enhanced uptake of fluorescent intensity of controls in DCs after stimulation with NP complex for 3 h. In contrast, PS and HH2 synergistically enhanced approximately 12.5 times higher mean fluorescence intensity than NY-ESO-1 alone. Efficient cross-priming has physiological importance for the initiation of cellular immune responses against tumors in the absence of the classical MHC-I presentation pathway [1]. Thus, co-localization of NY-ESO-1 and intracellular organelles in DCs was analyzed using confocal microscopy. As shown in Figure 6B, there was significantly more NY-ESO-1 adjuvant with PS or HH2 processed in compartments expressing LMP-2 compared with the control group. Co-localization experiment demonstrated that NY-ESO-1 localized to LMP-2/ LAMP-1-expressing compartments was predominantly in the NPH group versus other groups. PS-HH2 complex adjuvant could significantly enhance the NY-ESO-1 uptake and cross-presentation of DCs. Furthermore, we investigated the effect of PS-HH2 complex adjuvant on the maturation of DCs. Treatment of DCs with PS-HH2 for 24h resulted in an up-regulation of surface maturation markers, including CD80, CD86, and MHC- II (Figure 6C).

### Alum and PS-HH2 synergistically activate the Syk-PI3K-NF-κB signaling pathway for DC activation

**Figure 7:**
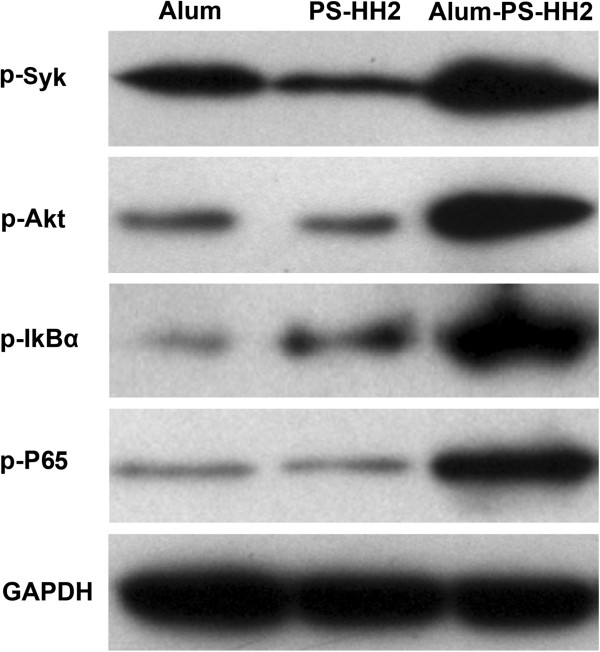
**Alum and PS-HH2 synergistically triggers activation of the NF-κB signal pathway.** Immature DCs derived from bone marrow were incubated and harvested on Day 5 for Western blotting. After treatment with alum (125 μg/ml), PS-HH2 (PS: 20 μg/ml and HH2: 40 μg/ml), or alum-PS-HH2 complex for 0, 5, 10 and 30 min, respectively. DCs were washed, lysed and used for Western blot analysis using antibodies to phospho-Syk, phospho-Akt, phospho-IkBα and phospho-NF-κB, respectively. GAPDH was detected on the same membrane and used as the loading control.

To further understand the molecular mechanisms involved in adjuvant complex-induced DCs, we examined the activation of Syk-PI3K/Akt signaling pathway by adjuvant complex stimulation. As shown in Figure 7, the phosphorylation of Syk and Akt was increased markedly in DCs after 15-min stimulation with APH complex, compared with alum or PS-HH2. Alum and PS-HH2 stimulation for 30 min synergistically triggered the activation of NF-κB, as assessed by the phosphorylation level using the phosphor-specific antibodies to IkBα and NF-κB. Both Syk and PI3K/Akt act as upstream regulators on NF-κB activation. Therefore, alum and PS-HH2 synergistically activates the Syk-PI3K-NF-κB signaling pathway.

## Discussion

Anticancer vaccines have been studied for decades, however, to date, the clinical effects of these vaccines remain limited. The development of vaccines has been hampered by the paucity of vaccines that are able to elicit an antitumor immune response to eliminate human tumors safely and effectively. Furthermore, the ability to sustain an effective antitumor immune response through repeated immunizations may be an important feature of therapeutic anticancer vaccines [34,35]. Recombinant adenoviral vector encoding the full sequence of NY-ESO-1 cDNA has been developed with some success [36,37]. However, the NY-ESO-1 DNA vaccine could not be used repeatedly due to the induction of anti-vector neutralizing antibodies. Thus, improvements are required. Adjuvant systems capable of enhancing the antigen specific immune response provide an attractive alternative strategy.

Here, for the first time, we present evidence that the APH complex adjuvant significantly enhanced the immunogenicity of NY-ESO-1 protein. The APH complex adjuvant could promote NY-ESO-1 uptake and maturation of DCs. Subsequent maturation of NAPH vaccine-pulsed DCs resulted in efficient targeting of full-length NY-ESO-1 proteins in the MHC-I pathway for cross-presentation to antigen-specific CD8^+^ T cells.

The synergetic effect on the enhancement of anti-tumor immune response among alum, PS and HH2 might be attributed to the following aspects: alum vaccination resulted in an increase in IL-4-producing cells to promote Th2-type immune responses [38]. Several studies have demonstrated that alum triggers lysosomal membrane damage with subsequent activation of the Nlrp3 inflammasome for its adjuvanticity [39,40]. Simultaneously, alum engages lipid rearrangements of DCs, resulting in an aggregating response of ITAM-Syk-PI3K-mediated phagocytes [41]. Alum can also accelerate the transmission of admixed antigen through the plasma membrane of DCs [41]. PS isolated from *E. coli*, a member of β-glycan, has a potent adjuvanticity to stimulate innate immunity and modulate the adoptive immune response. β-glycans are capable of priming/activating the iC3b receptor of neutrophils, macrophages, and NK cells for cytotoxicity against iC3b-opsonized tumor cells dependent on the CR3-Syk-PI3K pathway [25]. Our study demonstrated that alum and PS synergistically could activate the Syk-PI3K signaling pathway. HH2, a short synthetic innate defense-regulator peptide based on bovine bactenecin, is chemotactic for immature DCs, monocytes, neutrophils, and macrophages, and subsequently activates the MAPK-NF-κB pathway to up-regulate genes involved in innate immunity, such as IL-8 and MCP-1 [31]. The upstream tyrosine kinase Syk has an important role in NF-κB-mediated inflammatory responses. Therefore, alum, PS and HH2 might synergistically activate the NF-κB signaling pathway to enhance the antitumor immune response. Furthermore, due to its cationic property, HH2 facilitates antigen transport, uptake and presentation by antigen-presenting cells (APCs) [30]. Importantly, HH2 can enhance and modulate the adoptive immune response. However, the synergetic mechanism of the adjuvants remains to be further studied.

The generation of tumor-specific CTLs is well correlated with the prevention and eradication of malignant cells in tumor models. Vaccine-induced antibodies promote CD8^+^ T cell cross-priming via antibody-aided cross-presentation. Antibody-aided cross-priming of tumor specific CTLs could also contribute to the antitumor effect. Thus, an integrative weapon to recruit all available components of the immune system should be explored [42]. Our data has demonstrated that the NAPH vaccine could effectively stimulate NK-cell activity against MHC-I-deficient tumor. The NY-ESO-1-specific antibody titers and antibody-dependent cytotoxicity were evidently induced after NAPH vaccinations. In particular, the anti-serum obtained from the NAPH group could mediate tumor regression in a nude mouse model of human melanoma via adaptive transfer therapy. Furthermore, antitumor CD8^+^ CTLs were efficiently reactivated with the help of CD4^+^ T cells after NAPH immunization. The antitumor activity could be contributed to integrative immunoregulation, such as activation of NK cells, increase in adaptive T-cell responses, elevation in neutralizing antibody specific for NY-ESO-1, induction of ADCC activity against antibody-coated tumor cells, enhancement of Th1-type cytokine secretion (IFN-γ), and accentuation of CTL activity. Thus, NAPH may be exploited into a potent anticancer vaccine that could stimulate innate immune response and trigger the arms of the adaptive immune response, thus significantly inhibiting the tumorigenesis and growth of melanoma.

NY-ESO-1 is a promising tumor-associated target for vaccine-based immunotherapy of cancers. It is a non-mutated self-antigen that if frequently expressed in various types of human tumors, including melanoma, prostate, breast, lung, and transitional cell bladder carcinoma [43]. Furthermore, NY-ESO-1 has been found to share 94% identity with another cancer-testis antigen LAGE-1, at the nucleotide level. Although they are frequently co-expressed, 45% of melanoma patients have tumors expressing either one or the other antigen [44]. It has been reported that the NY-ESO-1 vaccine could also induce CD8^+^ T cells to kill tumor cells expressing LAGE-1 [45]. Thus, the NY-ESO-1 vaccine may serve as a common vaccine targeting many types of tumors.

In conclusion, here we show a new safe anti-tumor vaccine that could effectively induce innate immunity and NY-ESO-1-specific adoptive immune responses to inhibit the tumorigenesis and growth of melanoma. The APH complex adjuvant is promising as a novel immunomodulatory agent to mediate and enhance antigen-specific immune responses to prevent tumor refractory and treat cancer.

## Materials and methods

### Mice

Female C57/B6 mice (4-6 weeks old) were purchased from HuaFukang Biological Technology Company (Beijing, China) and maintained at the Animal Center of State Key Laboratory of Biotherapy of Sichuan University for 2 weeks of adaptive feeding prior to the start of experiment. The experimental protocol was approved by the Ethics Review Committee for Animal Experimentation of Sichuan University.

### Tumor cells

B16-F10 melanoma cells (ATCC, Rockville, USA) were transfected with the constructed plasmid pcDNA3.1 /NY-ESO-1 using Lipofectamine 2000 (Invitrogen, Carlsbad, CA). Next, the cells were grown with DMEM supplemented with 10% fetal bovine serum (FBS) (Gibco, Grand Island, USA) containing 800 μg/ml of G418 (Sigma, St. Louis, MO). B16 clones stably expressing NY-ESO-1 (NY-ESO-1-B16) were generated by limit-dilution cloning, which were confirmed by RT-PCR and Western blotting. Tumor cells were cultured and inoculated into mice if the viability was greater than 96%. All cell lines were free of mycoplasma infection. The ability of NAPH-immunized mice to reject NY-ESO-1-B16 melanoma *in vivo* was independently assessed in the protective and therapeutic models.

### *In vitro* cytokine secretion

To examine the effect of PS-HH2 combination on cytokine secretion, a constant amount of PS (10 μg) was combined with increasing concentrations of HH2, and used to treat PBMCs, followed by the chemokine/cytokine induction assay. Briefly, PS and HH_2_ were mixed with five ratios of PS: HH_2_ ranging from 4:1 (wt/wt) PS: HH_2_ to 1:4 (wt/wt) PS: HH_2_. Venous blood samples from healthy volunteers were collected and separated by centrifugation over a Ficoll Paque Plus (GE Healthcare Bio-sciences Corp., Piscataway, NJ, USA). PBMCs (5 × 10^5^) were seeded into a 24-well plate at 1 × 10^6^ cells/ml and stimulated with various PS-HH2 complexes, or the components alone. Following the stimulation for 24 h, the supernatants were collected and monitored for IFN-γ, TNF-α and MCP-1 levels using ELISA. In addition, the cytotoxicities of the PS-HH2 formulations were examined by monitoring the release of hemoglobin from red blood cells and LDH from human PBMCs as previously described by Kindrachuk et al. [30].

### Vaccines and immunization protocols

The expression and purification of NY-ESO-1 were prepared by the following steps. Briefly, hNY-ESO-1 (Invitrogen, Carlsbad, CA) was engineered into a bacterial expression plasmid PET32a (Invitrogen, Carlsbad, CA). The *E.coli* strain BL21 (DE3)-bearing recombinant PET32a-NY-ESO-1 was induced with IPTG for protein production. The crude NY-ESO-1 protein was expressed as soluble fusion constructed with N-terminal thioredoxin-His6-EK protease site tag (Trx-NY-ESO-1). The bacteria were lysed using a high-pressure homogenizer (APV 2000, Lubeck, Germany). NY-ESO-1 was purified via four process steps, which included Ni-chelating Sepharose affinity chromatography (GE Healthcare, Piscataway, NJ), excision of the Trx-His6-tag, removal of the Trx-His6-tag with second Ni-chelating Sepharose affinity chromatography, and Q-ion-exchange chromatography (GE Healthcare, Piscataway, NJ). The endotoxin level of the final purified NY-ESO-1 protein was measured using the QCL-1000 Endpoint Chromogenic Limulus Amebocyte Lysate (LAL) Assay kit (Lonza, Basel, Switzerland).

To prepare vaccines, PS (20 Sigma, St. Louis, MO) was first formulated with HH2 (Ketai Biotechnology Company, Shanghai, China) and alum (Brenntag Biosector, Frederikssund, Denmark) at 37°C for 10min, followed by the addition of NY-ESO-1, in which the endotoxin levels were approximately 0.02EU/μg. Briefly, 20 μg PS was mixed with 40 μg HH2, and then combined with 125 μg alum, followed by mixing with 5 μg recombinant NY-ESO-1 protein in PBS with a total volume of 100 μl. Components were omitted at the appropriate steps for the single- and two-component formulations.

In the preventive model, C57/B6 mice (n = 12) were vaccinated subcutaneously (s.c.) 3 times (on Days 0, 14, and 28) with NA, NAP, NAH, or NAPH. The mice treated with PBS were used as controls. Two weeks after the third immunization, the mice were challenged with NY-ESO-1-B16 cells. Briefly, the tumor cells (2 × 10^5^) were injected s.c. on the back and tumor volume was measured every 3 days when mice showed mammary neoplasia. Tumor volume was calculated by the following formula: tumor volume = 0.5 × length (mm) × [width (mm)]^2^.

In the therapeutic model, mice were inoculated s.c. with 2 × 10^5^ NY-ESO-1-B16 cells in 0.1 ml serum-free medium on Day 0. Once the tumor became palpable (>50 mm^3^), the mice were randomly divided into groups (12 mice/group) and immunized 3 times (on Days 5, 12 and 19) using the above-mentioned vaccines. The tumor size and body weight were measured as described above.

### Detection of antibody titer and antibody-dependent cellular cytotoxicity

Seven days after the third immunization, serum was collected and NY-ESO-1 specific antibody was determined using ELISA as previously described [46]. Briefly, plates (Nunclon, Roskilde, Denmark) were coated with NY-ESO-1 protein (0.1 μg/well) overnight at 4°C followed by blocking with 5% non-fat dry milk and 0.05% Tween-20 in PBS. Next, sera were incubated at different dilutions and goat anti-mouse IgG (1:3000 dilution; Zhongshan, Beijing, China), anti-mouse IgG2a, or anti-mouse IgG1 conjugated with horseradish peroxidase (1:400 dilution; Southern Biotech, Birmingham, AL). After the peroxidase color reaction was developed with TMB, the plates were read on an ELISA reader at A450.

Antibody binding to natural NY-ESO-1 expressed by NY-ESO-1-B16 melanoma cells was further assessed using a flow cytometry-adapted methodology previously reported by Piechocki et al. [47]. Briefly, 3 × 10^5^ NY-ESO-1-B16 cells were fixed and permeabilized with ice-cold 100% methanol for 10min. After incubation with diluted (1:400, 1:800, 1:1600, and 1:3200) mouse serum for 1 h at 4°C, the samples were washed with phosphate buffered saline (PBS), stained with FITC-conjugated anti-mouse IgG (BD Pharmingen, San Jose, CA) for 30min at 4°C, washed, and analyzed using BD FACS calibur flow cytometry (BD Biosciences).

To determine antibody-dependent cellular cytotoxicity, 1 × 10^6^ NY-ESO-1-B16 cells were incubated with pooled sera at 1:20 dilution, and labeled with Na^51^CrO_4_ for 1 h at 37°C. After the unbound antibodies and excess chromium were removed, the cells (1 × 10^4^) were incubated with mouse splenocytes obtained from naïve or treated mice for 4 h at 37°C. Finally, cell-free supernatant was collected and the release of chromium was measured using a gamma counter (LKB Wallac, Turku, Finland). The percent lysis was determined according to the formula: (experimental lysis-spontaneous lysis)/(maximum lysis – spontaneous release) × 100.

### NK assay and CTL

The activity of NK cells was determined using a classic NK assay against YAC-1 cells as previously described [48]. Briefly, YAC-1 cells were labeled with Na^51^CrO_4_, washed, and incubated with splenocytes obtained from naïve or treated mice 48 h after the first immunization for 4 h at 37°C.

One week after the third immunization, splenocytes were isolated, sensitized with NY-ESO-1 protein (10 μg/ml for 16 h at 37°C), and incubated with Na^51^CrO_4_ labeled NY-ESO-1-B16 cells at serial Target: Effector ratios (1:10, 1:20, and 1:40). The supernatants were harvested, and the released radioactivity was measured using a gamma counter (LKB Wallac, Turku, Finland).

### IFN-γ intracellular staining and ELISPOT assays

For intracellular staining of IFN-γ in CD4^+^ and CD8^+^ T cells, splenocytes were isolated one week after the last immunization, re-stimulated with NY-ESO-1 (10 μg/ml) for 1 h at 37°C, incubated for an additional 6 h in Golgi Plug and harvested, followed by staining with PerCP-anti-mouse CD4 and PE-anti-mouse CD8α. After fixation and permeabilization using a Cytofix/Cytoperm Kit, intracellular staining was achieved with FITC-anti–mouse IFN-γ (all from BD Bioscience/Pharmingen) according to the manufacturer’s instructions. After several washes, the cells were analyzed using BD FACS Calibur flow cytometer.

Splenocytes were harvested from naïve or immunized mice one week after the final immunization, and ELISPOT assays were performed using the mouse IFN-γ/IL-4 Dual-Color ELISpot kit (R & D systems, Minneapolis, USA) according to the manufacturer’s instructions. Briefly, 5 × 10^5^ splenocytes were stimulated with 10 μg/ml NY-ESO-1 in 100 μl/well RPMI 1640 supplemented with 10% FBS at 37°C for 48 h. Next, the plates were developed using an enzyme-linked colorimetric assay. The spots were quantified using an ELISPOT plate reader and software (Beijing Dakewe Biotech Company, Beijing, China).

### Adoptive transfer studies

One week after the third immunization, splenocytes and serum were isolated aseptically from naïve or treated C57/B6 mice for passive serum/cellular therapy. Next, 2 × 10^7^ donor splenocytes were injected i.v. into recipient mice for 2 consecutive days from the first day after tumor inoculation, as described [49]. For serum adoptive therapy, tumor-bearing mice were injected i.v. with 0.1 ml of the serum from Days 1 to 10 after the NY-ESO-1-B16 implantation.

To study of the passive serum therapeutic effect on human melanoma, female nude mice (5-7 weeks old) received s.c. inoculations of 5 × 10^6^ A375 cells on Day 0. Tumor-bearing mice were subjected to passive serum therapy, as described above.

### HE staining and immunohistochemistry

Tumor specimens, as well as heart, liver, spleen lung and kidney samples were fixed in 4% paraformaldehyde, and dehydrated by a series of graded alcohol solutions. Paraffin sections (5 μm) were prepared, and stained with H & E. For immunohistochemistry of tumor samples, slides were deparaffinized with xylene and rehydrated through graded alcohol solutions. Antigen retrieval was performed using steamed heat and endogenous peroxidase was quenched by 30 min incubation in 3% H_2_O_2_. Sections were separately incubated with anti-CD4, anti-CD8 and anti-CD57 mAbs (1:100 dilution; Abcam, Cambridge, MA) overnight at 4°C. Sections were then incubated with biotinylated anti-mouse antibody and streptavidin-biotinylated peroxidase complex. The peroxidase color reaction was developed with DAB chromogen. Finally, all sections were counterstained with Meyer’s hematoxylin. Infiltrated lymphocytes were observed and quantified under a microscope at 400× magnification. Intensity of lympocyte infiltration in the tumors was determined by the mean positive cell counts in the dermis around the NY-ESO-1-B16 tumor per field (10 randomly selected high power fields/slide). For each site, 3 pathologists performed a blind read of the glass slides.

### Antigen uptake and cross-presentation of DCs stimulated by the PH complex

Immature DCs derived from bone marrow were isolated from mice as previously described [50]. The phenotype of DCs was analyzed using a BD FACS Calibur flow cytometer. Briefly, C57BL/6 bone marrow cells were isolated and cultured in the presence of murine rIL-4 (10 ng/ml) and rGM-CSF (10 ng/ml) (both from Sigma). Non-adherent and loosely adherent immature DCs were harvested on Day 5 for experiments.

NY-ESO-1 protein was labeled with green fluorescence using a Pierce NHS-Fluorescein Antibody Labeling Kit (Invitrogen) according to the manufacturer’s instructions. NHS-labeled NY-ESO-1 (10 μg/ml) was combined with PS (20 μg/ml), HH2 (40 μg/ml), or PH complex at 37°C for 15 min. DCs were plated at 2 × 10^5^ cells/well in a 24-well plate and stimulated with protein or protein adjuvant complex. After incubation at 37°C for 3 h, the cells were fixed with 4% paraformaldehyde and stained for DNA with DAPI. The NY-ESO-1 uptake by DCs was observed under a confocal microscope.

For the NY-ESO-1 internalization studies of DCs, NY-ESO-1 protein was labeled with red fluorescence using the Alexa Fluor 594 protein labeling kit (Invitrogen) according to the manufacturer’s instructions. DCs were incubated with Alexa Fluor-labeled NY-ESO-1 or NY-ESO-1/complex adjuvant for 3 h at 37°C. After incubation, the cells were fixed in 4% paraformaldehyde and permeabilized in 0.1% Triton X-100/PBS. After Fc blocking with anti-mouse CD16/CD32 (BD Bioscience), the cells were separately stained with mouse FITC-anti-LAMP1 for late endosomes/lysosomes or FITC-anti-LMP2 antibody (both from BD) for proteasomes. Finally, DNA was stained with DAPI. The fluorescence intensity was calculated using the Image Pro Plus 6.0 software (Media Cybernetic, Inc. Silver Spring, USA). The background levels were determined by averaging the fluorescence intensity of controls. The mean fluorescence intensity was calculated for each photo and normalized by dividing the intensity by the number of cells. Significance was considered if the fluorescence intensity was 3 times higher than the other group.

After 24 h incubation with NY-ESO-1 or NY-ESO-1/adjuvant formulation, the phenotype of DC was analyzed using triple-color staining. The PERCP-anti-CD80, FITC-anti-CD86, PE-anti-MHCII were obtained from BD Biosciences PharMingen (San Diego, CA). Briefly, after antigen or antigen/adjuvant stimulation, the cells were first incubated with anti-mouse CD16/CD32 (BD Bioscience) to prevent nonspecific binding of mAbs to Fcγ receptors. Next, the DCs were stained with fluorochrome-conjugated antibodies. After washing, cells were analyzed using a BD FACS Calibur flow cytometer.

### Western blotting

Immature DCs derived from bone marrow were incubated and harvested on Day 5 for Western blot analysis. After treatment with alum (125 μg/ml), PS-HH2 (PS: 20 μg/ml, HH2: 40 μg/ml), or alum-PS-HH2 complex for 0, 5, 10, and 30 min, respectively, DCs were washed twice with Tris buffered saline (TBS) and lysed with RIPA buffer. The protein concentration was determined using the BCA assay. Equal amount of protein were separated by SDS-PAGE and transferred on polyvinylidene fluoride membranes (Millipore, Billerica, MA). After blocking with 5% non-fat milk /TBS-T (TBS containing 0.1% Tween 20), the blots were probed overnight at 4°C with antibodies to phospho-Syk, phospho-Akt, phospho-IkBα and phospho-NF-κB antibodies (all from Cell Signaling Technology, Beverly, MA), respectively. Blots were developed using a Supersignal West Pico chemiluminescent substrate kit (Pierce, Rockford, IL), following incubation with appropriate horseradish peroxidase-conjugated secondary antibody. GAPDH was detected on the same membrane and used as the loading control.

### Statistical analysis

Data were analyzed using the SPSS statistical software (IBM, Chicago, USA). Multiple group comparisons were performed using one-way analysis of variance followed by Tukey’s multiple-range testing. All values are depicted as the means ± SD. *P* < 0.05 was considered statistically significant.

## Abbreviations

*E.coli*: *Escherichia coli*; PS: Polysaccharide; s.c.: Subcutaneous; AP: Alum-polysaccharide; APH: Alum-polysaccharide-HH2; NAPH: NY-ESO-1-alum-polysaccharide-HH2; NAP: NY-ESO-1-alum-polysaccharide; NA: NY-ESO-1-alum; NAH: NY-ESO-1-alum-HH2; PH: Polysaccharide-HH2; TNF-α: Tumor necrosis factor α; IFN-γ: Interferon γ; MCP-1: Monocyte chemotactic protein-1; DCs: Dendritic cells; IDRs: Innate defense regulators; PBMCs: Peripheral blood mononuclear cells; ADCC: Antibody-dependent cell-mediated cytotoxicity; CTL: Cytotoxic T lymphocytes; TILs: Tumor infiltrating lymphocytes; MHC: Major histocompatibility complex; LDH: Lactate dehydrogenase; HE: Hematoxylin-eosin staining; SFCs: Spot-forming cells; RT-PCR: Reverse transcription polymerase chain reaction; IL-4: Interleukin-4; IL-8: Interleukin-8.

## Competing interests

The authors declare that they have no competing interests.

## Authors’ contributions

The authors’ contributions to this work are reflected in the order, with the exception of XC and LY who supervised the research and finalized the report. ML, HS and YM carried out the majority of the *in vitro* studies and all of the *in vivo* experiments. ML drafted the manuscript. YW, HZ and LL contributed to analysis and interpretation of data. Other authors participated in the design and coordination. All authors read and approved the final manuscript.

## Supplementary Material

Additional file 1: Figure S1 The effects of different PS-HH2 formulations on human PBMCs. A-C, PS and HH2 were complexed with five ratios of PS: HH2 ranging from 4:1 (wt/wt) PS: HH2 to 1:4 (wt/wt) PS: HH2. Human PBMCs (1×10^6^ cells/ml) were stimulated with PS, HH2, or PS-HH2 formulations for 24 h. Next, IFN-γ (A), TNF-α (B), and MCP-1 (C) in the culture supernatants was determined. *P < 0.05; **P < 0.005. D and E, Minimal cytotoxicity assay of PS-HH2 formulations. Human red blood cells (D) or PBMCs (E) were stimulated with various PS-HH2 complexes, or the components alone. Following stimulation, the supernatants were collected and measured for the release of hemoglobin or LDH. D: Total hemoglobin release from red blood cells induced by PS-HH2 complexes. E: LDH release from PBMCs following stimulation with PS-HH2 complexes. All data were representative of at least 3 independent experiments. Click here for file

Additional file 2 **Tumor inhibition rate to all formations and mean tumor volume of all groups for each of the in vivo experiments. Table S1.** The inhibition ratio (%) was calculated by the following formula: inhibition ratio (%)= [(A-B)/A]×100, where A is the average tumor weight of the control group, and B is the tumor weight of the treated group. **Table S2.** The mean tumor volume of all groups for each of the in vivo experiments. Click here for file

Additional file 3: Figure S2 Body weight and histology of major organs. A, Body weight. Body weight was measured every 3 days during the entire treatment. B, Sections of heart, liver, spleen and kidney were stained with H&E. There was no obvious histological difference between the groups (magnification, 200×). Click here for file
