# Supplementary figures and images for: Effective inhibition of melanoma tumorigenesis and growth via a new complex vaccine based on NY-ESO-1-alum-polysaccharide-HH2
Source: Mol Cancer. 2014 Jul 28;13:179. doi: 10.1186/1476-4598-13-179 (PMC4120012; doi:10.1186/1476-4598-13-179)

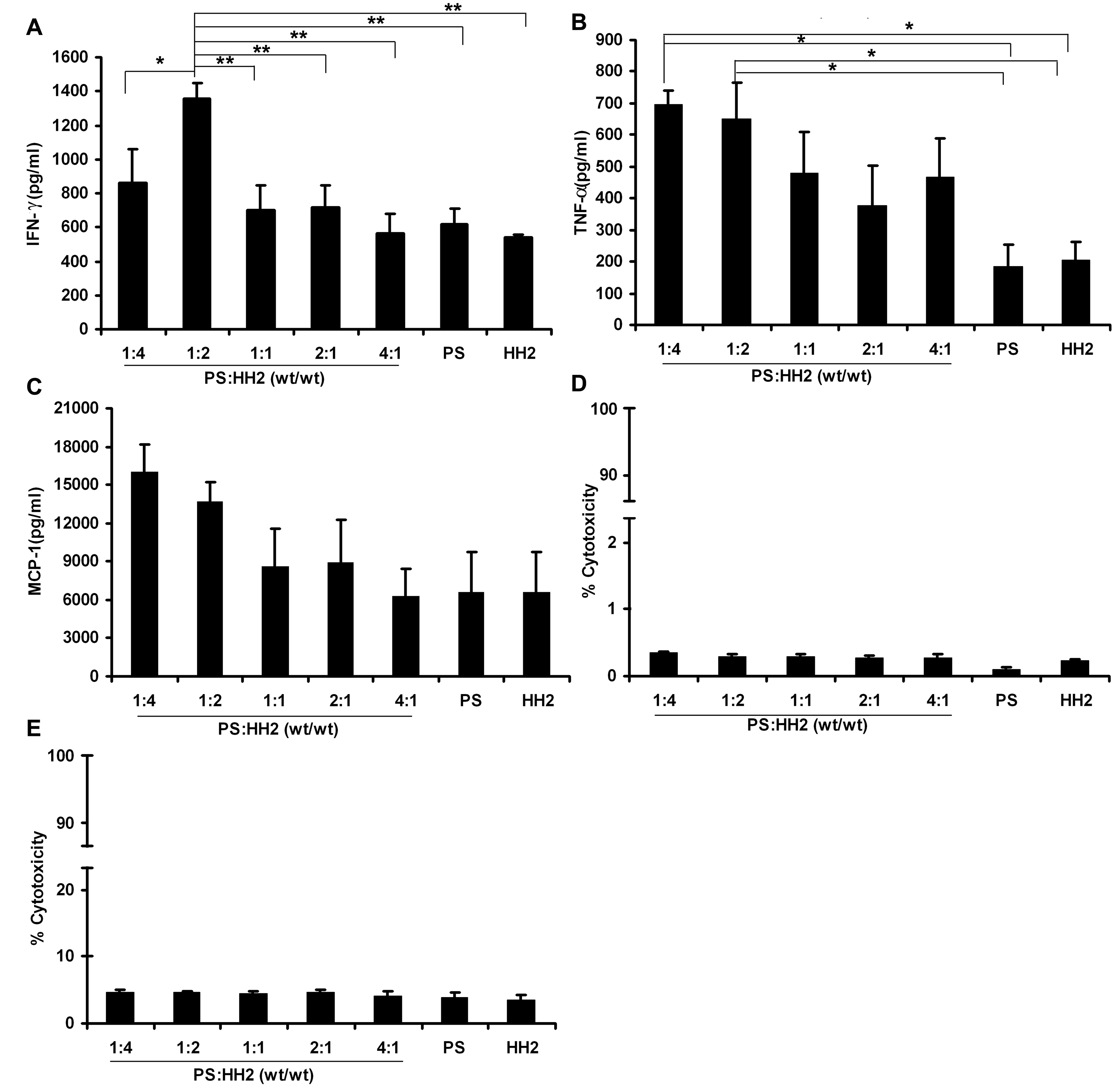

Supplement: Additional file 1: Figure S1 — The effects of different PS-HH2 formulations on human PBMCs. A-C, PS and HH2 were complexed with five ratios of PS: HH2 ranging from 4:1 (wt/wt) PS: HH2 to 1:4 (wt/wt) PS: HH2. Human PBMCs (1×10^6^ cells/ml) were stimulated with PS, HH2, or PS-HH2 formulations for 24 h. Next, IFN-γ (A), TNF-α (B), and MCP-1 (C) in the culture supernatants was determined. *P < 0.05; **P < 0.005. D and E, Minimal cytotoxicity assay of PS-HH2 formulations. Human red blood cells (D) or PBMCs (E) were stimulated with various PS-HH2 complexes, or the components alone. Following stimulation, the supernatants were collected and measured for the release of hemoglobin or LDH. D: Total hemoglobin release from red blood cells induced by PS-HH2 complexes. E: LDH release from PBMCs following stimulation with PS-HH2 complexes. All data were representative of at least 3 independent experiments. [file 1476-4598-13-179-S1.tiff]

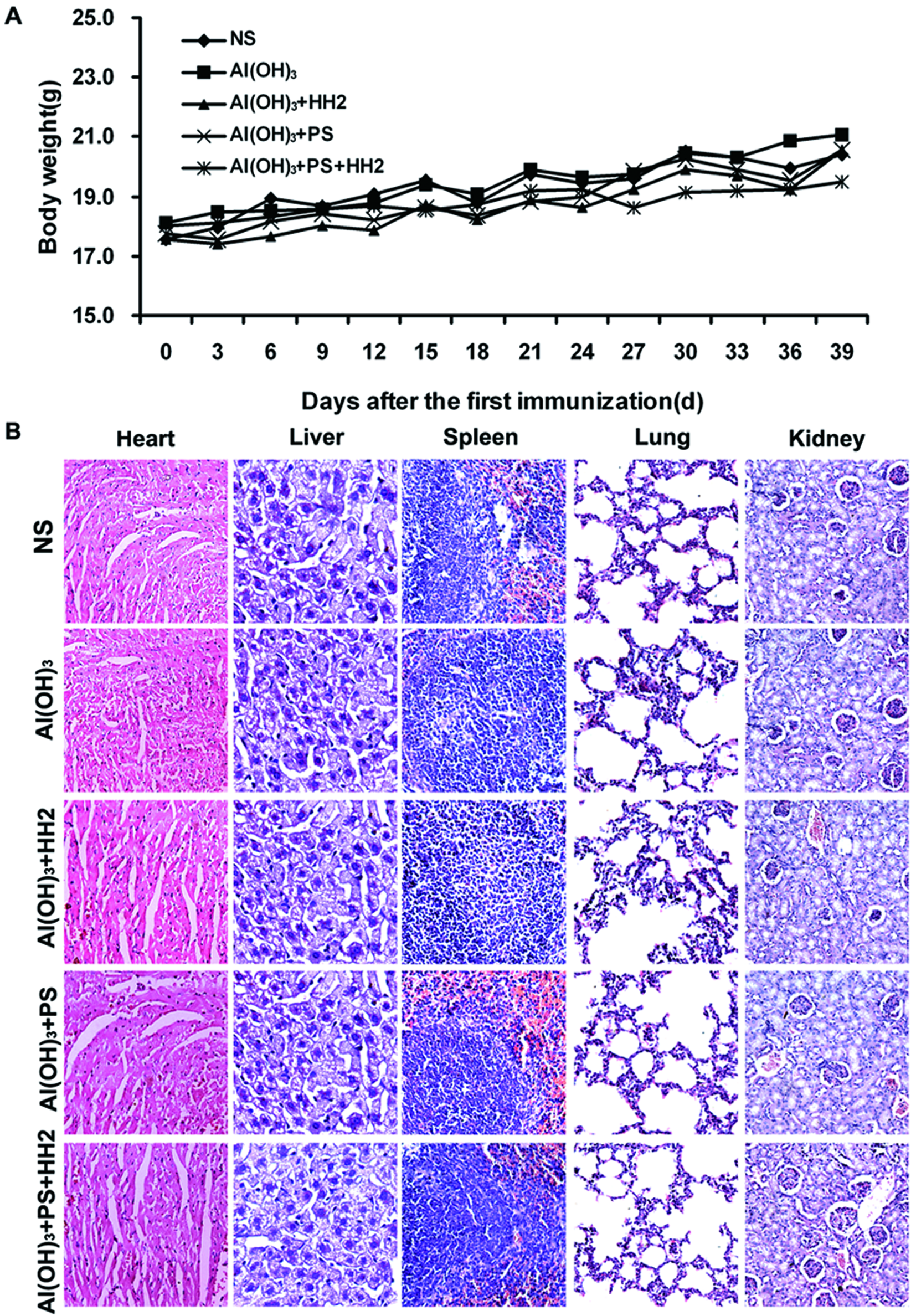

Supplement: Additional file 3: Figure S2 — Body weight and histology of major organs. A, Body weight. Body weight was measured every 3 days during the entire treatment. B, Sections of heart, liver, spleen and kidney were stained with H&E. There was no obvious histological difference between the groups (magnification, 200×). [file 1476-4598-13-179-S3.tiff]
