# Supplementary material for: Effective inhibition of melanoma tumorigenesis and growth via a new complex vaccine based on NY-ESO-1-alum-polysaccharide-HH2
Source: Mol Cancer. 2014 Jul 28;13:179. doi: 10.1186/1476-4598-13-179 (PMC4120012; doi:10.1186/1476-4598-13-179)
Supplement: Additional file 2 — Tumor inhibition rate to all formations and mean tumor volume of all groups for each of the in vivo experiments. Table S1. The inhibition ratio (%) was calculated by the following formula: inhibition ratio (%)= [(A-B)/A]×100, where A is the average tumor weight of the control group, and B is the tumor weight of the treated group. Table S2. The mean tumor volume of all groups for each of the in vivo experiments. [file 1476-4598-13-179-S2.doc]

**Supplemental table 1: Tumor inhibition rate to all formations for each of the *in vivo* experiments.**

| **Groups** | **Prophylactic model (%)** | **Therapeutic model (%)** | **Adoptive cellular therapy model (%)** | **Adoptive serum therapy model (%)** | **Adoptive serum therapy nude mice model (%)** |
| --- | --- | --- | --- | --- | --- |
| **Al(OH)_3_** | **44.40** | **14.50** | **23.90** | **19.50** | **N.D.** |
| **Al(OH)_3_+HH2** | **48.90** | **41.00** | **30.30** | **13.50** | **42.20** |
| **Al(OH)_3_+PS** | **54.70** | **52.50** | **31.20** | **26.20** | **47.50** |
| **Al(OH)_3_+PS+HH2** | **79.00** | **68.20** | **52.50** | **70.70** | **78.40** |

**The inhibition ratio (%) was calculated by the following formula: inhibition ratio (%)= [(A-B)/A]×100，where A is the average tumor weight of the control group, and B is the tumor weight of the treated group.**

**Supplemental table 2: mean tumor volume of all groups for each of the *in vivo* experiments.**

| **Groups** | **Prophylactic model (mm^3^)** | **Therapeutic model (mm^3^)** | **Adoptive cellular therapy model (mm^3^)** | **Adoptive serum therapy model (mm^3^)** | **Adoptive serum therapy nude mice model (mm^3^)** |
| --- | --- | --- | --- | --- | --- |
| **NS** | **1674.56±474.6** | **2431.06±704.12** | **2699.07±1145.49** | **2439.39±1915.99** | **1521.05±855.25** |
| **Al(OH)_3_** | **931.11±257.14** | **2079.62±864.79** | **2052.84±489.18** | **1963.6±871.48** | **N.D.** |
| **Al(OH)_3_+HH2** | **855.18±131.21** | **1434.47±560.31** | **1879.97±199.53** | **2110.8±1912.33** | **879.74±386.20** |
| **Al(OH)_3_+PS** | **759.4±158.12** | **1155.18±439.41** | **1858.10±56.64** | **1801.15±863.55** | **799.31±83.59** |
| **Al(OH)_3_+PS+HH2** | **351.8±167.47** | **773.06±633.73** | **1282.32±338.4** | **715.41±234.04** | **328.15±83.59** |
